# Synaptic plasticity, neural circuits, and the emerging role of altered short-term information processing in schizophrenia

**DOI:** 10.3389/fnsyn.2014.00028

**Published:** 2014-11-25

**Authors:** Gregg W. Crabtree, Joseph A. Gogos

**Affiliations:** ^1^Department of Physiology and Cellular Biophysics, College of Physicians and Surgeons, Columbia UniversityNew York, NY, USA; ^2^Department of Neuroscience, College of Physicians and Surgeons, Columbia UniversityNew York, NY, USA

**Keywords:** neuropsychiatric diseases, short-term synaptic plasticity, neural circuits, 22q11.2 microdeletion, DISC1, schizophrenia, autism spectrum disorder, risk genes

## Abstract

Synaptic plasticity alters the strength of information flow between presynaptic and postsynaptic neurons and thus modifies the likelihood that action potentials in a presynaptic neuron will lead to an action potential in a postsynaptic neuron. As such, synaptic plasticity and pathological changes in synaptic plasticity impact the synaptic computation which controls the information flow through the neural microcircuits responsible for the complex information processing necessary to drive adaptive behaviors. As current theories of neuropsychiatric disease suggest that distinct dysfunctions in neural circuit performance may critically underlie the unique symptoms of these diseases, pathological alterations in synaptic plasticity mechanisms may be fundamental to the disease process. Here we consider mechanisms of both short-term and long-term plasticity of synaptic transmission and their possible roles in information processing by neural microcircuits in both health and disease. As paradigms of neuropsychiatric diseases with strongly implicated risk genes, we discuss the findings in schizophrenia and autism and consider the alterations in synaptic plasticity and network function observed in both human studies and genetic mouse models of these diseases. Together these studies have begun to point toward a likely dominant role of short-term synaptic plasticity alterations in schizophrenia while dysfunction in autism spectrum disorders (ASDs) may be due to a combination of both short-term and long-term synaptic plasticity alterations.

## Introduction—neuropsychiatric disease manifestations and hypotheses of neural circuit dysfunction

The recent advances over the past several decades in molecular biology, human genomics, and bioinformatics has helped to enable the identification of numerous candidate genes and distinct genetic mutations in a variety of human neuropsychiatric illnesses that may be the responsible agents for causing their symptomology. Based on the genetic mutations identified by human genomic analyses, many of these mutations have been introduced into the mouse genome to create genetic mouse models of these human diseases in the hope that the invasive and precisely refined experimentation permitted in mice will help to reveal the details of the molecular and neuronal dysfunction underlying these diseases. Despite the availability of these powerful experimental tools, the daunting molecular, anatomic, and functional complexity of the central nervous system demands that targeted and well-considered hypotheses be generated within this overwhelming complex system in order to direct experimental investigations to the systems and processes where dysfunction is most likely to be found in each unique disease.

When considering the underlying mechanistic causes of the dysfunction and symptomology of neuropsychiatric diseases, a thoughtful consideration of the most prominent features and most characteristic symptoms of each disease is a useful starting point for hypothesis generation. It has been proposed that the core commonality of dysfunction amongst neuropsychiatric diseases is a deficit in the information processing performed both by and between the various neural microcircuits located throughout the brain (Yizhar et al., 2011; Uhlhaas and Singer, 2012). Although multiple parameters work together to determine the computations performed by neural circuits, the synaptic plasticity that dynamically regulates synaptic function and hence the information flow between neurons at each of the millions of synapses throughout a neural microcircuit is thought to play a fundamental role in this microcircuit computation (Abbott and Regehr, 2004). While short-term synaptic plasticity leads to short-term changes in synaptic function (lasting from milliseconds to minutes) that regulate the moment-to-moment information flow through a neural circuit, long-term synaptic plasticity leads to persistent changes in synaptic function (lasting from hours to the lifetime of the synapse) that fundamentally and adaptively alters microcircuit function in response to activity indicating changing computational demands (Zucker and Regehr, 2002; Collingridge et al., 2010; Granger and Nicoll, 2013). Due to this critical role in controlling microcircuit computation, here we consider dysfunction in both long-term and short-term synaptic plasticity mechanisms as promising candidates underlying the neural microcircuit dysfunction that is likely responsible for neuropsychiatric disease symptomology (Table 1). While dysfunction in distinct neuropsychiatric diseases may result from alterations in either or both short-term and long-term synaptic plasticity, as a paradigm, studies of schizophrenia—where short-term synaptic plasticity alterations dominate—serve as an illustrative example of how targeted hypotheses of synaptic plasticity dysfunction can be formulated by beginning with the temporal nature of disease manifestations as a strategic starting point.

**Table 1:** **Synaptic plasticity mechanisms and information flow**.

**Synaptic plasticity**	**Cellular mechanism involved**	**Duration of functional change**	**Affect on information flow**
*Short-term*	NT-release pathways (Pr)	Transient	**Inherent synaptic dynamics** that normally determine moment to moment synaptic/network information flow
	NT-receptors	Short-lived	
Presynaptic depression	Reduced presynaptic NT release	ms – minutes	Time-dependent filtering:
	High initial Pr, NT release		Postsynaptic neuron receives LP filtered information from presynaptic neuron
	↓Pr – ↑NT-vesicle depletion (↓RRP)		Onset/change/transient detection
Presynaptic facilitation	Increased presynaptic NT release	ms – minutes	Time-dependent filtering:
	Low initial Pr, NT release		Postsynaptic neuron receives HP filtered information from presynaptic neuron
	↑Pr – ↑Residual presynaptic Ca^2+^		Burst/rate detection
Postsynaptic depression	Reduced postsynaptic NT responses	ms – minutes	Similar to presynaptic depression
	NT-receptor saturation/desensitization		
Augmentation PTP	Increased NT release, ↑Pr	10 s of seconds	Allows maintenance of information flow during periods of high activity that would otherwise be reduced by ST depression
	Unresolved: ↑RRP, prolonged residual	10 s of seconds – mins	
	Ca^2+^, ↑Ca^2+^ flux, Ca^2+^ sensitivity of NT release		
*Long-term*	NT-receptors NT-release (Pr)	Persistent, long-lived	**Transiently induced**, long-lived changes synaptic function that fundamentally and persistently alter synaptic/network computation
	Structural changes	Hours – lifetime	
LTD-presynaptic	Reduced presynaptic NT release	Persistent, hours+	Changes ST synaptic dynamics
	Decreased initial Pr		Shifts ST plasticity toward more facilitating, HP filtering
LTD-postsynaptic	Reduced postsynaptic NT response	Persistent, hours+	Decreases postsynaptic gain
	↓NT-receptor number/function		Time-independent, decreases information flow at all frequencies
LTP-presynaptic	Increased presynaptic NT release (increased initial Pr)	Persistent, hours+	Changes ST synaptic dynamics Shifts
			ST plasticity toward more depressing, LP filtering
LTP-postsynaptic	Increased postsynaptic NT response	Persistent, hours+	Increases postsynaptic gain
	↑NT-receptor number/function		Time-independent, increases in information flow at all frequencies

*Summary of short-term and long-term synaptic plasticity mechanisms and their impact upon synaptic and network information flow. Abbreviations: NT, neurotransmitter; Pr, the probability of neurotransmitter release; RRP, readily releasable pool of NT-vesicles; LP, low-pass filtering, the preferential transmission of information to the postsynaptic neuron at lower presynaptic activity rates; HP, high-pass filtering, the preferential transmission of information to the postsynaptic neuron at higher presynaptic activity rates; ST, short-term; LT, long-term; PTP, post-tetanic potentiation; LTP, long-term potentiation; LTD, long-term depression*.

Schizophrenia is a relatively common and severely debilitating psychiatric illness that is characterized by a somewhat variable yet core set of clinical symptomology. Although the most striking features of schizophrenics are their disorganized thinking and speech, delusions, and hallucinations (so-called positive symptoms of psychosis), the disease is also characterized by both “negative” symptoms (e.g., social withdrawal, blunted emotional range, lack of motivation, anhedonia) as well as severe cognitive deficits that may in fact contribute more substantially to functional disability and quality of life than the more prominent positive symptoms (Ranganath et al., 2000; Carter et al., 2008; American Psychiatric Association, 2013).

Current drug therapies, while substantially efficacious in treating positive symptoms, show little benefit in correcting the more disabling cognitive and negative symptoms and disease-preventative measures are currently non-existent (Buchanan et al., 2007; Brown and McGrath, 2011). Schizophrenia research targeted at understanding the precise molecular and neuronal mechanisms underlying disease dysfunction represents the most promising path toward more efficacious therapy and cure. To achieve these ends, genetic mouse models based upon robust candidate schizophrenia susceptibility genes identified by human genomic studies have recently been employed. These genetic mouse models are currently the best available analytical platform as they permit the invasive, high-resolution studies—impossible to conduct in human subjects—necessary to identify and elucidate the precise disease mechanisms present in neuropsychiatric diseases at the neuronal and molecular level (Arguello et al., 2010). At present, combining thoughtful consideration and analysis of human symptomology and the findings from mouse models based upon bone fide schizophrenia genetic risk variants has begun to suggest that, while long-term synaptic plasticity mechanisms may be relatively intact, there is an important and dominant role of short-term synaptic plasticity dysfunction in schizophrenia pathogenesis (Arguello and Gogos, 2012).

Although the clinical presentation and evolution of the schizophrenia can be highly variable, cognitive impairment frequently pre-dates diagnosis which often initially presents with the psychotic positive symptoms while negative symptoms usually become more prominent only at later times (O'Carroll, 2000). This evolution of symptomology strongly suggests that cognitive and psychotic symptomology may represent the primary disease process and be fundamental to understanding disease mechanisms. Significantly, both the specific cognitive deficits and psychotic features characteristic of schizophrenia suggest a fundamental problem with the moment-to-moment information processing performed by neural circuits within the brain (Compte et al., 2000; Mongillo et al., 2008). The cognitive deficits in schizophrenia are both pervasive and profound and range from impaired sensory processing to deficits in the cognitive control mechanisms necessary to manage and organize information (Barch, 2009; Dias et al., 2011; Lesh et al., 2011). Although schizophrenics show deficits in both working memory (a short-term memory process) and episodic memory (involving both short- and long-term processes), performance testing in human subjects suggests that both of these deficits likely involve an underlying deficit in short-term information management as episodic memory deficits can be overcome by employing certain encoding strategies indicating that long-term recall processes are largely intact (Holthausen et al., 2003; Ragland et al., 2005; Bonner-Jackson et al., 2008; Van Snellenberg, 2009).

The psychotic features of schizophrenia likewise point toward a deficit in the short-term processing of information. The nature of disorganized speech, hallucinations, and delusions all point to a moment-to-moment deficit in the instantaneous processing of on-going streams of information rather than a deficit involving long-term processes. Moreover, the drug-responsiveness of psychosis—as well as the ability of certain drugs and medications to induce psychosis in healthy individuals—further suggests a likely subtle defect in the dynamics of short-term information processing rather than a significant derangement or degeneration of microcircuit connectivity or architecture (Arguello and Gogos, 2012). Thus, taken together, both the psychosis and the nature of the cognitive deficits in schizophrenia strongly suggest pathologic alterations heavily weighted toward dysfunction in short-term information processing dynamics by neural microcircuitry. Given that the short-term, ongoing information processing performed by neural circuits is believed to be largely dictated by the short-term synaptic plasticity that controls the instantaneous dynamics of synaptic information flow strongly suggests that the neural circuit dysfunction in schizophrenia may to a large extent result from underlying alterations in short-term synaptic plasticity (Abbott and Regehr, 2004; Mongillo et al., 2008; Deng and Klyachko, 2011). Consistent with this hypothesis, studies in genetic mouse models of schizophrenia have found that the cognitive and synaptic plasticity alterations observed in these mice are dominated by deficits in working memory and short-term synaptic plasticity rather than in episodic memory and long-term synaptic plasticity (Koike et al., 2006; Kvajo et al., 2008; Stark et al., 2008; Sigurdsson et al., 2010; Drew et al., 2011b; Fénelon et al., 2011, 2013; Arguello and Gogos, 2012).

While at a biological top level it would seem necessary that disease dysfunction must follow gene dysfunction, the lack of full penetrance of schizophrenia within pedigrees—most notably the only 50% concordance between monozygotic twins—suggests that while gene dysfunction clearly plays a prominent role, other factors are also significantly contributing to disease genesis (Karayiorgou and Gogos, 1997). There is broad supporting data that environmental factors act both in concert with genetic background as well as epigenetically to enhance risk for schizophrenia (Prasad et al., 2010). Significant environmental risk factors predisposing to schizophrenia may include—but are not limited to—a variety of chronic, early-life social, emotional, and metabolic stressors, perinatal viral illness and hypoxic insults, and chronic, early-life use of some drugs of abuse, most significantly cannabis (Vilain et al., 2013; Schmitt et al., 2014). Despite the importance of identifying these risk factors, both risk genes and environmental factors still remain at a frustrating distance from a detailed, mechanistic understanding by which they lead to the deranged information processing at the level of the neural circuit dysfunction underlying disease (Lisman et al., 2008; Hu et al., 2010; Sigurdsson et al., 2010; Steullet et al., 2010; Arguello and Gogos, 2012; Filipović et al., 2013; Spellman and Gordon, 2014).

Although the molecular and neuronal dysfunction that leads to psychosis and schizophrenia remains poorly understood, theories of dysregulation of neurotransmitter systems including hyper-function of dopaminergic systems and hypo-function of glutamatergic systems (principally NMDA receptors) have been proposed as candidates underlying altered synaptic plasticity (Blum and Mann, 2002; Baldessarini and Tarazi, 2006). These theories, which were largely developed prior to the genomic era and were originally based upon clinical observations of responses to various drugs and medications that either precipitated or ameliorated psychosis, have been heavily investigated and at times supported by findings in a variety of models of schizophrenia (Mohn et al., 1999; Lahti et al., 2001; Dalmau et al., 2007; Manahan-Vaughan et al., 2008; Wiedholz et al., 2008; Ecker et al., 2009; Belforte et al., 2010; Carlén et al., 2012; Inta et al., 2014). But whether manipulations of these neurotransmitter systems simply phenotypically mimic the symptoms of schizophrenia or whether alterations in these NT systems are truly fundamental to the human disease process still remains unknown. Intriguingly, however, the genetic risk variants identified in most genomic studies have only rarely been found to be among those genes comprising the principal components of these neurotransmitter systems which suggests that if alterations of these NT systems are fundamentally involved in schizophrenia they must be so indirectly through the downstream actions of risk genes (Glatt et al., 2003). Thus, while it would likely be unwise not to explore the function of these NT systems in mouse models based upon human genetic risk variants, it may also be equally unwise to view these models without more unbiased eyes. We would suggest a broader and more measured investigative approach centered upon mouse models based upon bone fide genetic risk variants as a means to approach neural circuit dysfunction in disease though investigative analysis of the substrates most fundamentally involved in determining neural circuit function, principally synaptic plasticity and neural circuit architecture. By taking advantage of the unbiased nature of identifying genetic risk variants as a gateway to unravel disease-relevant neural circuit dysfunction, this approach may likely be the most fruitful and efficacious means to reveal the functional and molecular details of disease mechanisms required to create the most effective, targeted therapeutics.

This example of the hypothesis development and experimental approach for schizophrenia thus serves as a paradigm for the effective approach to the study of other neuropsychiatric diseases by showing how high-risk susceptibility genes found through human genomic studies can be used to develop genetic mouse models that not only recapitulate the behavioral deficits found in human disease but that further allow detailed analysis of the neuronal, synaptic, and neural circuit dysfunction underlying these behavioral alterations. As neural circuit dysfunction is a common underlying feature in neuropsychiatric disease, a research strategy focusing upon assessing the function of distinct neural circuits implicated in disease symptomology and the neuronal and synaptic mechanisms that underlie their function will likely provide the optimally productive approach for revealing the fundamental molecular mechanisms underlying neuropsychiatric disease (Lisman et al., 2008; Akil et al., 2010; Arguello and Gogos, 2012). While the unique symptomology of schizophrenia strongly suggests that neural circuit dysfunction may be predominantly due to alterations in short-term synaptic plasticity, the varied symptomology within the spectrum neuropsychiatric diseases can likewise be used to guide targeted experiments assessing the unique contributions of short-term and long-term synaptic plasticity mechanisms underlying neural circuit dysfunction in distinct neuropsychiatric diseases.

## Potential causes of neural circuit dysfunction in neuropsychiatric disease

The commonality of dysfunction among the psychiatric diseases is a failure of the brain to process information appropriately in order to produce a normal and appropriate response to the demands of a given task or situation. While the specific details of how the brain accomplishes the processing of information remain understood at only the most rudimentary level, it seems apparent that the informational computation performed by neural circuits is critical to the interpretation of the world around us that ultimately leads to adaptive and advantageous behavioral responses (Uhlhaas and Singer, 2006, 2012; Yizhar et al., 2011). Although this review focuses exclusively upon the role of synaptic plasticity alterations in neuropsychiatric disease and their roles in neural microcircuit dysfunction, it is useful to first acknowledge and consider alternative paths that can also lead to the outcome of network dysfunction in neuropsychiatric disease. Neural microcircuit function is principally determined by three factors: (1) the network architecture that is defined by the synaptic connectivity between the neurons within a microcircuit, (2) the synaptic plasticity which dynamically regulates the strengths of these individual synapses and ultimately controls the information flow between the neurons in the network, and (3) the postsynaptic ensemble integration of these synaptic inputs that determines whether synaptic information received from presynaptic neurons successfully leads to an AP in the post-synaptic neuron. Thus, in considering potential neuronal causes of neural circuit dysfunction, it is possible to envision a comprehensive yet manageable set of pathologic scenarios that could lead to impaired neural circuit function in neuropsychiatric disease.

In perhaps the simplest scenario, the architecture and connectivity of the neural circuit could be normal and the intrinsic function of the neurons and synapses within the circuit could be normal while some outside factor could be causing neural circuit dysfunction. That both electrolyte imbalances as well as exogenous drugs and medications can alter neuronal function and acutely cause psychiatric symptomology suggests that similar mechanisms could play causal roles in neural circuit dysfunction in chronic neuropsychiatric disease (Webb and Gehi, 1981; Morton, 1999; Turjanski and Lloyd, 2005). Both the dopaminergic agonists and NMDA receptor antagonists that precipitate acute psychotic states that mimic the psychosis of schizophrenia appear to operate by this mechanism in the context of presumably otherwise normal basal neural circuit function (Lahti et al., 2001; Manahan-Vaughan et al., 2008; Ecker et al., 2009).

In another scenario in which neural circuit architecture is normal, alterations in either neuronal or synaptic function could lead to pathological alterations in neural circuit performance. Changes in either the excitability of the neurons within the circuit or the regulation by synaptic plasticity of the strengths of the synapses that define the functional connectivity of the circuit will alter the information flow through the circuit and thus lead to disruptions of neural circuit computation (Matveev and Wang, 2000; Zheng et al., 2004; Deng and Klyachko, 2011; Rotman et al., 2011; Yizhar et al., 2011; Ransdell et al., 2012). Current paradigms of schizophrenia and autism implicating the prominent alterations in synaptic plasticity observed in mouse models based on identified human genetic risk variants suggest that such functional changes alone could be largely responsible for neural circuit dysfunction (Markram and Markram, 2010; Arguello and Gogos, 2012).

In a complementary scenario in which both neuronal and synaptic function are normal, alterations in neural circuit architecture alone could similarly lead to alterations in the information processing performed by neural circuits. The neuron to neuron connectivity created by the synapses between neurons is a critical determinant in neural circuit function and alterations in this circuit architecture would have a significant impact upon information processing. Such alterations in connectivity in neural circuits likely play a central role in the neural circuit dysfunction in neurodegenerative diseases in which neuronal death and the inability to grow and maintain new synaptic connections would be expected to significantly alter neural circuit architecture (Hensch, 2005; Le Be and Markram, 2006; Pratt et al., 2008; Ricoy et al., 2011). Both the prominent progressive synaptic and neuronal degeneration observed in Alzheimer disease and the emerging role of deficient adult neurogenesis in major depression suggest that alterations in neural circuit architecture and the dynamic maintenance of that architecture may alone play substantial roles in neural circuit dysfunction (Selkoe et al., 2012; Miller and Hen, 2015).

In a final scenario, it is possible to have information processing abnormalities in the context of both normal neural circuit architecture and normal neuronal and synaptic function if the long-range connectivity between distinct neural circuits in different brain regions is altered. As complex behaviors and decisions frequently require the transmission of information processed in a neural circuit in one brain region to another circuit located in a distant brain region, the appropriate functional connectivity between these distinct neural circuits is critical to effective global information processing within the brain (Achard et al., 2006; Wang, 2010; Modha and Singh, 2010). Suggesting an important causal role in neuropsychiatric pathophysiology, prominent alterations in long-range connectivity between brain regions have long been implicated in major depression and have also more recently been implicated in schizophrenia, autism, and OCD (Lawrie et al., 2002; Nestler et al., 2002; Belmonte et al., 2004; Meyer-Lindenberg et al., 2005; Geschwind and Levitt, 2007; Murias et al., 2007; Welch et al., 2007; Krishnan and Nestler, 2010; Lynall et al., 2010; Markram and Markram, 2010; Sigurdsson et al., 2010; Ahmari et al., 2013; Brennan et al., 2013; Uhlhaas, 2013).

Furthermore, apart from the distinct scenarios presented above, it is possible—if not likely—that the information processing deficits present in neuropsychiatric illnesses could be caused by neural circuit dysfunction that is due to a combined dysfunction of the mechanisms presented in these individual scenarios. Despite this diversity of mechanisms potentially responsible for neural circuit dysfunction, here in this review we focus solely upon the possible role of alterations in synaptic plasticity as contributing to the dysfunction of information processing by neural circuits in neuropsychiatric diseases. Alterations in synaptic plasticity mechanisms represent particularly attractive candidates for information processing dysfunction in neuropsychiatric disease as their impact upon the dynamics of information flow through neural circuits appears particularly critical to the computation performed by these circuits (Abbott and Regehr, 2004; Mongillo et al., 2008; Deng and Klyachko, 2011). Although a detailed understanding of the information processing performed by neural circuits currently remains out of reach, convergent results from experimental and theoretical studies strongly suggest that synaptic plasticity—and in particular short-term synaptic plasticity—is fundamental in governing these computations (Markram and Tsodyks, 1996; Tsodyks and Markram, 1997; Milner et al., 1998; Dobrunz and Stevens, 1999; Martin et al., 2000; Abbott and Regehr, 2004; Whitlock et al., 2006; Mongillo et al., 2008; Deng and Klyachko, 2011).

In addition to synaptic plasticity mechanisms, however, there are many other forms of plasticity within neural systems that can also act upon neural circuits to modify their function. The growth of new synaptic connections, the birth of new neurons via neurogenesis that then become incorporated into existing circuitry, the modulatory impact of glial cells acting either alone or in concert with neurons, and the actions of neurotrophic factors released by either neurons or glia can all lead to significant changes in the function and architecture of neural circuits and have all been implicated in contributing to dysfunction in neuropsychiatric diseases (Gottschalk et al., 1998; Gu, 2002; Lamprecht and LeDoux, 2004; Ghashghaei et al., 2006; Le Be and Markram, 2006; Reif et al., 2006, 2007; Todd et al., 2006; Mao et al., 2009). As a comprehensive consideration of these diverse mechanisms is beyond the scope of this review, however, herein we limit our consideration exclusively to neuronal aspects of synaptic plasticity mechanisms that act acutely in a synaptic activity-dependent manner to modify the function of pre-existing synapses within neural circuits and the potential role of alterations in these mechanisms in the circuit dysfunction in neuropsychiatric disease.

## Synaptic plasticity as a vantage point for psychiatric disease

While there still remains a daunting knowledge gap between the symptomology of neuro-psychiatric disease and the causes of this symptomology at the synaptic, neuronal, and neural circuit level, considering how distinct forms of neuronal dysfunction might be reflected in disease symptomology can be cautiously applied toward hypothesis generation that can lead to targeted experiments in understanding the underlying dysfunction in these diseases. Broadly, neuropsychiatric diseases with both profound and persistent symptomology—such as the more severe neurodevelopmental and neurodegenerative disorders—suggest a more fundamental and pervasive underlying neuronal derangement while those diseases with more nuanced and episodic symptomology might suggest nearly normal neuronal, synaptic, and neural circuit function with dysregulation occurring at the level of the fine-tuning and modulation of neuronal, synaptic, and neural circuit function (Table 1).

In considering the cognitive deficits present in Alzheimer's disease (AD), for example, while there is some waxing and waning of symptomology, the memory deficits characteristic of AD dementia are largely persistent, pervasive, and progressive. While these memory deficits are possibly due in part to deficits of long-term plasticity, the major pathology underlying the dementia is likely to be predominantly the consequence of the neuronal death and degeneration that would fundamentally alter the connectivity of neural circuits involved in memory formation and storage (rather than due to alterations in the cellular mechanisms involved in LTP *per se*). The plausibility of this interpretation is supported by the broadly reported findings indicating that neuronal degeneration and death, pervasive alterations in neuronal and synaptic morphology, and deficits in synaptic maintenance are fundamental characteristics of AD pathology. Thus, while the memory deficits of AD do indeed imply neuronal and neural circuit dysfunction, these dysfunctions are likely a consequence of significant alterations in functional connectivity between neurons that affect neural circuit architecture rather than modulatory changes in neuronal function that serve to sculpt information processing in a largely intact and functional neural circuit. Taken together, both the symptomology and the profound degeneration and neuronal derangements present in AD dementia suggest that the underlying disease dysfunction in most cases is not likely primarily due to subtle changes in neuronal function such as alterations in synaptic plasticity mechanisms.

In contrast to the case of AD, the nature of the psychotic symptomology of both schizophrenia and bipolar disorder suggests the likelihood of a much more nuanced neuronal dysfunction that leads to information processing dysfunction. The severity of psychotic symptomology in these diseases tends to be much more episodic in nature suggesting periods of relatively normal neuronal and neural circuit function. This episodic nature coupled with the frequent responsiveness of psychosis to medication supports a much more subtle alteration in information processing, possibly in the context of both nearly normal neuronal and neural circuit function. Further supporting the “nearly normal” hypothesis of psychosis are the many drugs that can acutely lead to transient psychotic symptomology in healthy individuals that is clinically indistinguishable from the psychosis in psychiatric disease. As these drugs can induce psychosis in individuals with presumably normal baseline neuronal and neural circuit function, it is likely that psychosis in psychiatric disease can manifest in the context of relatively minor perturbations in neuronal function. The moment-to-moment information processing deficits that appear to be characteristic of psychosis suggest the possibility that short-term synaptic plasticity mechanisms—that control the computational rules within neural circuits—may represent the primary locus of dysfunction underlying this symptomology. In further support of likely short-term plasticity dysfunction in schizophrenia are the significant deficits in working memory in schizophrenia. While the exact neural substrates of working memory remain to be fully elucidated, both experimental as well as theoretical work suggest that working memory is supported largely by the short-term synaptic plasticity mechanisms at play within neural networks. Thus, both the psychotic features as well as the working memory deficits characteristic of schizophrenia strongly suggest that short-term synaptic plasticity mechanisms represent a promising target to explore for dysfunction, a notion supported by results from studies in mouse models based on valid schizophrenia risk genes which have most prominently displayed pronounced alterations in short-term synaptic plasticity (Arguello and Gogos, 2012).

With this framework in mind, we next examine the evidence for dysfunction in neuronal network performance and synaptic plasticity in neuropsychiatric diseases for which robust, corresponding genetic mouse models of disease have been created. Despite the recent explosive progress in the identification of an abundance of risk genes for various neuropsychiatric diseases, only a very small subset of these identified genetic variants have been found to be highly penetrant with respect to their ability to reliably lead to disease. As such, the creation of etiologically valid mouse models of disease based upon high-risk, highly penetrant genetic variants has only been possible to date in a small subset of neuropsychiatric diseases. Based upon these considerations, we limit our consideration here to two diseases with the strongest genetic mouse models: schizophrenia and autism.

## Schizophrenia

Schizophrenia is a heterogeneous disorder with an approximate prevalence of 1% in most populations that most frequently presents in late adolescence to early adulthood and has a strong genetic component based upon the increased incidence observed in twin studies and within in familial pedigrees (McGuffin et al., 1995; Karayiorgou and Gogos, 1997; Sullivan et al., 2003; Lichtenstein et al., 2009a). Schizophrenia is characterized by positive symptoms (disordered thought and speech, delusions, and hallucinations—classic psychotic features), negative symptoms (blunted emotional range, anhedonia, social withdrawal, decreased motivation), and cognitive deficits (deficits in executive function, attention, and both episodic and working memory) that together define the illness (O'Carroll, 2000; Forbes et al., 2009; American Psychiatric Association, 2013). While the causes of most cases of schizophrenia remain unknown, a combination of genetic and environmental factors appears to collaborate in its pathogenesis. A wide array of schizophrenia candidate risk genes and genetic mutations have been identified—from both rare, syndromic and familial cases as well as through large genomic association studies—and have implicated genes with diverse function including those that are either known to or have since been shown to affect synaptic function (Frankle et al., 2003; Gogos and Gerber, 2006; Lisman et al., 2008; Xu et al., 2008, 2011, 2012; Stefansson et al., 2009; Drew et al., 2011a; Girard et al., 2011; Ripke et al., 2011, 2013; Rodriguez-Murillo et al., 2012). While a subset of genomic studies has suggested that genetic risk variants may be potentially biased toward postsynaptically expressed genes, mouse models based upon the most relevant and highly penetrant risk variants have most often shown alterations in short-term synaptic plasticity, which strongly implicates a presynaptic site of dysfunction (Kirov et al., 2012; Fromer et al., 2014; Hall et al., 2014; but see Frankle et al., 2003; Xu et al., 2012).

Abnormalities in the functional connectivity between microcircuits in different brain regions have been put forth as an important pathophysiological mechanisms underlying dysfunction in schizophrenia and functional imaging and EEG studies in schizophrenia as well as studies in mouse models support this possibility (Ford et al., 2002; Lawrie et al., 2002; Meyer-Lindenberg et al., 2005; Esslinger et al., 2009; Stephan et al., 2009; Sigurdsson et al., 2010; Brennan et al., 2013; Uhlhaas, 2013). Assessment of network function in schizophrenia with EEG has shown increased local microcircuit activity as evidenced by enhanced gamma-band oscillations under resting, unstimulated conditions (Kikuchi et al., 2011; Spencer, 2012). In contrast to the increased local microcircuit activity at rest, during a variety of cognitively demanding tasks local microcircuit activity is significantly reduced as indicated by a reduction in gamma oscillations during these tasks compared to healthy controls (Spencer et al., 2003; Ford et al., 2008; Hirano et al., 2008; Haenschel et al., 2009; Minzenberg et al., 2010). Furthermore, some of the observed network alterations have also been seen in both unaffected monozygotic twins and healthy first-degree relatives of schizophrenics suggesting that local circuit dysfunction may represent a significant intermediate functional endophenotype (Hong et al., 2008; Hall et al., 2011). Finally, *in vivo* plasticity studies conducted on schizophrenic sample groups have shown either reductions or complete absence of long-term plasticity evoked by transcranial stimulation indicating the possibility of significant impairments in long-term plasticity induction mechanisms (Fitzgerald et al., 2004; Daskalakis et al., 2008; Frantseva et al., 2008; Hasan et al., 2011, 2012a,b, 2013).

Despite the large number of schizophrenia risk genes and gene mutations implicated through various methodologies, only a subset of these targets appear to be able to confer by themselves a substantially increased risk of schizophrenia. Furthermore, amongst this subset the directionality and nature of the functional change (e.g., reduced expression or function vs. enhanced expression or function) caused by the genetic mutations that is required to create an accurate functional representation of human disease in mouse models is known for only a very small subset of these implicated genes. Here we discuss the findings in genetic mouse models of schizophrenia that meet these criteria. Intriguingly, studies among these mouse models have revealed that, while long-term synaptic plasticity is relatively intact, a striking preponderance of short-term plasticity alterations suggesting that the neural circuit dysfunction caused by these genetic risk variants may likely be due in large part to alterations in presynaptic function that fundamentally impact synaptic computation (Table 2).

**Table 2:** **Synaptic plasticity alterations in animal models of schizophrenia genetic risk variants**.

**Risk variant**	**Function/Location**	**Disease model**	**ST plasticity alterations**	**LT plasticity alterations**	**Network architecture alterations**	**Network function alterations**
22q11.2	Multiple	Loss of function	↑↑ST depression	↓LTP CA3-CA1	Probable:	Reduced synchrony between mPFC and dHPC. Long-range connectivity deficit
	Multiple		↓↓ST potentiation	↓LTP mPFC Layer V	Arborization	
			PPR – normal	Normal CA3-CA1 LTD	Synaptogenesis	
				Normal CA3-CA1 depotentiation		
PRODH	L-proline degradation	Loss of function	↓↓PPR – more depressing	↓LTP CA3-CA1 (presynaptic)	Unexplored	NA
	Mitochondrial		↑↑Pr			
DGCR8	miRNA processing	Loss of function	↑↑ST depression	Normal LTP CA3-CA1	Possible:	NA
	Nuclear		↓↓ST potentiation	Normal LTP mPFC-V	Arborization	
			PPR – normal		Synaptogenesis	
DISC1	Unresolved, Multiple	Loss of function	↓ST potentiation	Normal LTP DG-CA3	Probable:	NA
	Cytosolic, intracellular	Gain of function?	↓↓ST freq facilitation	Normal LTP CA3-CA1	Arborization	
		Novel function?	↓↓PPR, ↑↑Pr		Synaptogenesis	
					Neurogenesis	
Calcineurin	SER/THR phosphatase	Loss of function	PPR, PTP – normal, HPC	↓LTD CA3-CA1	Probable:	↓γ-osc power mPFC
PPP3CC	Cytosolic		PPR, Pr – normal, mPFC	Normal LTP CA3-CA1	Gene expression	↑SWR events, HPC
			↑↑ST depression, mPFC	↓LTP threshold CA3-CA1	Structural plasticity	
			↑↑ST synaptic fatigue			Synaptogenesis
Neuregulin 1	Ligands for ERBB RTKs	Loss of function	PPR – Glu, normal	↑NRG1, ↓LTP	Highly probable:	γ-osc alterations
NRG1	Transmembrane	Gain of function	↓↓PPR – GABA, ↑↑Pr	↓NRG1, ↑LTP	Migration	
			↑PPR, ↑Pr, GABA			Synaptogenesis
RGS4	G-protein signaling, GAP	Loss of function	↑PPR, ↓Pr (RGS2)	↓LTD (presynaptic Pr)	Possible:	NA
	Cytosolic				Developmental expression	
Dysbindin	Unresolved, Multiple	Loss of function	↑PPR, ↓Pr (DM)	↑↑LTP	Unknown	NA
DTNBP1	Cytosolic		↑↑ST facilitation (DM)	LTD normal	Underexplored	
			↓Pr (↓RRP, ↓mEPSC mPFC)			
			PPR normal, CA3-CA1			
AKT1	SER/THR kinase	Loss of function	ST facilitation – normal (DM)	↓LTD (presynaptic, DM)	Possible:	NA
PKB	Cytosolic		ST depression – normal (DM)	↓LTP, CA3-CA1; PP-DG	Arborization	
			PTP – normal (DM)		Diverse signaling	
			↓↓ST potentiation			

*Summary of schizophrenia genetic risk variants and synaptic plasticity alterations observed in animal models. Abbreviations: PPR, paired-pulse ratio, a measure of short-term plasticity that compares the amplitude of the 2nd synaptic response in terms of the 1st synaptic response for two closely-timed evoked synaptic responses; mPFC, medial prefrontal cortex; mEPSC, miniature, spontaneous excitatory synaptic events; HPC, hippocampus; dHPC, dorsal hippocampus; CA3-CA1, Schaffer collateral synapses on to HPC CA1 neurons; DG-CA3, dentate gyrus mossy fiber synapses onto CA3 neurons; PP-DG, entorhinal cortical synapses onto dentate gyrus neurons activated through perforant-path stimulation; NA, not assessed; miRNA, micro RNA; SER/THR, serine/threonine; ERBB RTK, ErbB receptor tyrosine kinase; GAP, GTPase activating protein; DM, Drosophila animal model; Glu, L-glutamate; freq, frequency; γ-osc, gamma oscillations; SWR, sharp-wave ripple. For more detailed descriptions for these findings and associated references (see main text)*.

### The 22q11.2 microdeletion and related genes

Microdeletions of the 22q11.2 locus are among the most common chromosomal abnormalities, occur predominantly *de novo*, and account for 1–2% of sporadic schizophrenia cases (Karayiorgou et al., 1995, 2010; McDonald-McGinn et al., 2001; Xu et al., 2008). Individuals with the 22q11.2 microdeletion are characterized by a high incidence of emotional problems and a spectrum of cognitive deficits, and approximately 30% eventually develop schizophrenia or schizoaffective disorder in adolescence or early adulthood (Pulver et al., 1994; Bearden et al., 2001; Woodin et al., 2001; Sobin et al., 2005; Chow et al., 2006).

A mouse model has been created to recapitulate the human deletion syndrome by creating an analogous deletion in the mouse genome that spans a segment syntenic to the 1.5-Mb human 22q11.2 microdeletion that results in the heterozygous deletion of 27 genes (Stark et al., 2008). Behavioral analysis of this mouse indicated both cognitive deficits, as manifested by impaired spatial working memory and contextual and cued fear learning, as well as deficits in sensorimotor gating—paralleling findings in schizophrenia—as demonstrated by reductions in pre-pulse inhibition (PPI) of acoustic startle (Swerdlow and Geyer, 1998; Stark et al., 2008). To further refine the interpretation of behavioral assays, results from novel object recognition and latent inhibition behavioral assays—that require hippocampal function but are largely independent of PFC function—indicated that purely hippocampal-dependent behaviors were intact suggesting that the primary functional deficit in these animals was largely restricted to dysfunction within the PFC (Fénelon et al., 2013).

Electrophysiological assessment of the 22q11.2-DS equivalent mouse showed that while nearly all forms of short-term and long-term plasticity tested in the hippocampus were unaltered (with the exception of modest reductions in 100 Hz induced LTP at CA3-CA1 synapses), synapses onto layer V neurons in the mPFC—although showing normal basic synaptic transmission and paired-pulse ratios—demonstrated enhanced synaptic depression during high-frequency stimulus trains and reductions in the initial phase of the synaptic potentiation (short-term potentiation) induced by the high frequency stimulus train for LTP induction (Drew et al., 2011b; Fénelon et al., 2013). Finally, *in vivo* recordings from mice performing a spatial working memory task have shown that the functional, synchronous coupling between the microcircuits in the hippocampus and the PFC is significantly reduced during working memory performance in deletion mice suggesting that the working memory deficits observed in these mice might be related to this disconnectivity (Sigurdsson et al., 2010). Overall these findings of cognitive deficits in the face of largely normal hippocampal electrophysiological function suggest that the primary dysfunction in the deletion syndrome mouse is likely due to a primary functional deficit in PFC microcircuit function and the connectivity between the PFC and the hippocampus. In addition to these findings in mice that employ the full 27 gene microdeletion, in order to more precisely dissect the underlying dysfunction, further evaluations have been performed in mice in which only single genes from within this region have been deleted individually.

### DGCR8

DGCR8 is a gene within the 22q11.2 microdeletion whose gene product is involved with processing micro-RNAs (mi-RNAs) within the nucleus which are involved with repressing translation of their target mRNAs (Tomari and Zamore, 2005; Karayiorgou et al., 2010). While homozygous deletion of DGCR8 is lethal, heterozygous deletion such as occurs in the 22q11.2 deletion syndrome results in the reduction of numerous mi-RNAs leading to enhanced expression of their targets due to the loss of mi-RNA repression (Stark et al., 2008; Xu et al., 2013). Mice missing one copy of the DGCR8 gene (DGCR8± mice) demonstrate reduced PPI of acoustic startle, impaired performance in spatial working memory, but, in contrast to deletion-syndrome mice, both normal contextual and cued fear learning (Stark et al., 2008). Similar to electrophysiology findings in the deletion syndrome mouse, measures of both short-term and long-term plasticity were found to be normal at CA3-CA1 synapses in the hippocampus of DGCR8(±) mice, while synapses onto layer V neurons in the mPFC showed normal paired-pulse ratios (suggesting normal initial Pr) but enhanced synaptic depression during high-frequency stimulus trains and reductions in the initial phase of the synaptic potentiation induced by high frequency trains of stimuli (Fénelon et al., 2011). Thus, heterozygous deletion of DGCR8 alone seems to be able to account for most of the electrophysiological deficits found in the deletion syndrome mouse while the absence of fear-learning deficits suggests the involvement of other genes within the 27-gene deletion in these behaviors.

### PRODH

PRODH is a gene within the 22q11.2 microdeletion whose gene product is involved with L-proline degradation within mitochondria and its homozygous or heterozygous deletion results in a gene-dose dependent elevation in L-proline in both the periphery and within the CNS (Phang et al., 2001; Karayiorgou et al., 2010). Human subjects with hypomorphic mutations in their PRODH gene have a disease called hyperprolinemia type I that is characterized by seizures and significantly increased incidence of schizophrenia and schizoaffective disorder (Phang et al., 2001; Jacquet et al., 2005; Raux et al., 2007; Clelland et al., 2011). Mice with homozygous functional deletion of the PRODH gene (PRODH knockdown mice) demonstrate reduced PPI of acoustic startle, normal performance in spatial working memory tasks, and reductions in both contextual and cued fear learning (Gogos et al., 1999; Paterlini et al., 2005). Electrophysiological assessment of CA3-CA1 hippocampal synapses with field recordings in PRODH-deficient mice showed increased evoked EPSPs and reduced paired-pulse ratios (depressing compared to facilitating in control animals at a 20 ms interval) both of which suggest increased initial probability in NT release (Pr) in PRODH-deficient mice (Paterlini et al., 2005). These mice also showed decreased CA3-CA1 LTP induction which was notable for a complete absence of its presynaptic component whereas control animals showed increased Pr after LTP induction (Zakharenko et al., 2003; Paterlini et al., 2005). Taken together these electrophysiological results suggest that PRODH deficiency leads to altered presynaptic function with increased initial Pr and an inability of LTP induction to lead to further increases in initial Pr. Interestingly, PRODH-deficient also mice showed evidence of dopaminergic dysregulation within the PFC as evidenced by enhanced hyperlocomotion and dopamine release within the PFC upon amphetamine challenge as well as a possibly compensatory enhanced expression of the dopamine degradative enzyme COMT (a gene located within the 22q11.2 microdeletion that would be expected to be compromised in its ability to undergo an equal compensatory upregulation in patients with the microdeletion). Overall, the findings in PRODH-deficient mice suggest primarily alterations in presynaptic function that—despite normal working memory performance in these mice—would be expected lead to significant alterations in synaptic filtering and network computation within the hippocampus (Tsodyks and Markram, 1997; Abbott and Regehr, 2004). Furthermore, although the underlying mechanism remains to be resolved, PRODH hypofunction also appears to lead to dopaminergic dysregulation and studies in schizophrenics that have revealed a synergy between PRODH and COMT hypofunction support this as a potentially important disease mechanism (Raux et al., 2007).

### DISC1

Disrupted in schizophrenia 1 (DISC1) is a gene that was implicated in schizophrenia through its discovery as the gene disrupted by a chromosomal translocation that cosegregated with mental illness in a family pedigree prominently affected by schizophrenia, bipolar disorder, and major depression (St Clair et al., 1990; Millar et al., 2000; Blackwood et al., 2001). The precise cellular and biological role of the DISC1 gene product remains uncertain and resolution of this question has been hindered by the fact that its protein sequence appears to share no homology with other known proteins (Millar et al., 2000). To determine possible functions of Disc1, studies have identified numerous protein-binding partners of Disc1 the most promising of which suggest that Disc1 may play a significant role in modulating cellular function through alterations in cAMP levels and protein kinase function. Disc1 binds to and regulates the phosphodiesterase, PDE4b, and mice with a DISC1 mutation that models the consequences of the human chromosomal translocation demonstrate enhanced cAMP levels due to decrease PDE4 activity (Millar et al., 2005; Kvajo et al., 2011). Furthermore, Disc1 has also been shown to bind to and inhibit the function of the protein kinase GSK3β (Mao et al., 2009) leading to reduced phosphorylation of some of its protein targets. Although in early development Disc1 shows a broader expression pattern, in the adult brain prominent expression is restricted to the dentate gyrus and, to a lesser extent, the CA1 regions within the hippocampal microcircuitry (Austin et al., 2004). Based upon this expression pattern and given the potential role of adult neurogenesis in schizophrenia and other neuropsychiatric diseases, a number of studies have suggested a potentially important role for DISC1 in regulating microcircuit architecture through its regulation of neurogenesis in the dentate gyrus (Ghashghaei et al., 2006; Reif et al., 2006, 2007; Mao et al., 2009; Ming and Song, 2009; Inta et al., 2011; Kim et al., 2012). Within neurons, Disc1 has been shown to be present in multiple subcellular compartments including dendrites and dendritic spines as well as in growth cones and axonal terminals suggesting the possibility that Disc1 could regulate either postsynaptic or presynaptic function (Millar et al., 2005; Kirkpatrick et al., 2006; Taya et al., 2007; Bradshaw et al., 2008; Brandon and Sawa, 2011; Randall et al., 2014).

Using a genetic mouse model employing a DISC1 mutation that accurately recapitulates the consequences of the human chromosomal translocation, a comprehensive battery of behavioral analyses has revealed a pattern of cognitive deficits in these mice that appears to reflect cognitive deficits found in schizophrenia (Koike et al., 2006; Kvajo et al., 2008). Amongst this battery of cognitive behavioral assays, Disc1 mutant mice displayed an isolated deficit in spatial working memory as indicated by impaired performance on a delayed alternation t-maze task. Significantly, largely hippocampus-dependent behavioral tasks—contextual fear conditioning, novel object recognition, Morris water maze, and the eight-arm radial maze win-shift paradigm—all failed to reveal significant alterations in Disc1 mutant mice (Kvajo et al., 2008). Taken together, the results of these behavioral assays suggest an isolated deficit in the short-term maintenance and utilization of information—as opposed to information storage and retrieval—that likely results from information processing dysfunction in the PFC rather than in the hippocampus (Compte et al., 2000; Baddeley, 2012; Roux et al., 2012).

Electrophysiological assessment of granule cell mossy fiber synapses onto CA3 neurons—motivated by the high expression of Disc1 in the dentate gyrus—showed both normal basic synaptic transmission as well as normal LTP in response to 100 Hz stimulation (Austin et al., 2004; Kvajo et al., 2011). Measures of short-term plasticity at these synapses, however, revealed reduced paired-pulse ratios (suggesting increased Pr) and reductions in the short-term frequency facilitation characteristic of this synapse both pointing to selective deficits in short-term plasticity in Disc1 mutant mice (Nicoll and Schmitz, 2005). Electrophysiological assessment of CA3-CA1 hippocampal synapses, in contrast, showed both normal long-term and short-term plasticity with the exception of an isolated reduction in the initial, short-term synaptic potentiation induced by the 100 Hz stimulus train employed for LTP induction (Kvajo et al., 2008). Interestingly, this isolated deficit in short-term synaptic potentiation at CA3-CA1 synapses may potentially represent a significant pathophysiology for schizophrenia as a similar deficit in this short-term potentiation mechanism was observed at mPFC synapses in both the 22q11.2-DS equivalent mice and DGCR8 (+/−) mice (Fénelon et al., 2011, 2013). While assessment of the function of Disc1 at synapses within the PFC has not been reported in Disc1 mutant mice, a recent study employed *in utero* electroporation and optogenetic techniques to selectively examine the presynaptic role of Disc1 at synapses onto cortical layer II/III pyramidal neurons (Maher and LoTurco, 2012). This study provided evidence that Disc1 may positively regulate neurotransmitter release as Disc1 knockdown led to increased paired pulse ratios (consistent with reduced Pr) as well as disrupted kinetics of evoked synaptic responses potentially consistent with less synchronized NT release. Taken together, the results of these synaptic function studies suggest a limited role of Disc1 in long-term synaptic plasticity but a potentially significant role in regulating presynaptic short-term plasticity mechanisms that would be expected to significantly impact both synaptic and network computation (Abbott and Regehr, 2004).

### Calcineurin

Calcineurin dysfunction has been implicated in the pathophysiology of schizophrenia (Gerber et al., 2003; Horiuchi et al., 2007; Liu et al., 2007) with reduced expression or function as the most likely responsible disease mechanism (Eastwood et al., 2005). Calcineurin is a cytosolic Ca^2+^-dependent protein phosphatase that regulates many cellular processes throughout the body including the CNS where is has been shown to regulate various neuronal processes including synaptic plasticity (Groth et al., 2003). Calcineurin-deficient mice display a number of behavioral abnormalities relevant to schizophrenia including severe reductions in working memory performance and deficits in the PPI of acoustic startle (Zeng et al., 2001; Miyakawa et al., 2003). Furthermore, these studies in mice with a forebrain specific calcineurin deficiency have described altered synaptic plasticity and neural network performance suggesting that hypofunction of calcineurin in schizophrenia could likewise alter synaptic plasticity and lead to disruptions of normal information processing (Cottrell et al., 2013; Suh et al., 2013).

It was originally reported that forebrain calcineurin deficiency led most notably to severe working memory deficits as well as to deficits in LTD at Schaffer collateral (CA3)—CA1 hippocampal synapses (Zeng et al., 2001). This study further showed that the frequency dependence of the stimulus required to induce long-term plasticity was shifted in calcineurin-deficient mice such that LTP (vs. LTD) induction was favored at lower than normal stimulus frequencies suggesting that deficiencies in LTD were permissive of LTP induction. Significantly, the LTD deficiency appeared to be due to an alteration of a post-synaptic mechanism since, in the genetic model employed, calcineurin was not deficient in the CA3 presynaptic neurons as these synapses. In further support of a post-synaptic mechanism, presynaptic function and short-term plasticity were unaltered as evidenced by normal PPR and post-tetanic potentiation (PTP). Although this study failed to identify the precise functional defect that led to LTD deficiency, the normal AMPA-R and NMDA-R mediated synaptic responses observed in calcineurin-deficient mice suggested possible dysfunction in downstream postsynaptic LTD induction mechanisms.

A follow-up study by this same group has recently described apparent network dysfunction in the hippocampus of freely behaving calcineurin-deficient mice (Suh et al., 2013). While *in vivo* recording from dorsal CA1 showed normal place fields and place cell activation during exploratory behavior, during rest periods place cells were overactive and temporally disorganized in their activity during sharp-wave ripple events (SWRs). The authors suggest that, as SWRs during rest are believed to orchestrate sequential “replay” of place cell activity in order to maintain spatial information, the absence of sequential replay of place cells during SWRs observed in calcineurin-deficient mice may underlie the severe spatial WM deficit previously described (Zeng et al., 2001). Finally, in an effort to link synaptic plasticity alterations to network dysfunction, the authors speculate that the LTD deficit present in CA1 might be responsible for the enhanced excitability within this neural circuitry that appeared to underlie the lack of organized “replay” of place cells during SWRs in calcineurin-deficient mice. Taken together the results of these two studies suggest calcineurin deficiency in schizophrenia would most likely have little impact upon short-term plasticity and synaptic filtering but would disrupt the ability to sculpt information processing by neural circuits by limiting the ability of LTD to down-regulate synaptic gain post-synaptically in order to appropriately control network excitability.

In contrast to the possible role of calcineurin in modulating postsynaptic plasticity in the hippocampus, another recent study in calcineurin-deficient mice has reported deficits in presynaptic function within the PFC (Cottrell et al., 2013). Although the cortical layer and synapse under study was not precisely clarified, these synapses in the PFC showed normal PPR and normal synaptic depression during 20 Hz stimulation; however, during 40 Hz stimulus trains calcineurin-deficient mice showed enhanced synaptic depression. Additional experiments in cultured neurons employing synapto-pHluorins revealed impaired synaptic vesicle exocytosis-endocytosis cycling suggesting that the enhanced synaptic depression was due to augmented synaptic fatigue that limited sustained NT release. Parallel experiments at hippocampal mossy fiber–CA3 synapses—where calcineurin remained expressed postsynaptically—showed similar deficits in sustained NT release indicating that presynaptic deficiency of calcineurin was responsible for the observed deficit. Further studies indicated that calcineurin-deficient mice also demonstrated neural circuit dysfunction as evidenced by reduced power of γ-oscillations in both PFC slices and in *in vivo* PFC recordings from mice exploring a novel environment. As γ-oscillations in the PFC are believed to be critical for working memory and other executive functions and as these high-frequency oscillations rely heavily upon sustained, high-frequency NT release, the observed deficits in network function suggest the possibility that the MW impairments in calcineurin-deficient mice may ultimately result from presynaptic NT release dysfunction. Thus, the combined results of this study provide a possible mechanism sequentially linking presynaptic NT release dysfunction, to neural circuit dysfunction, to cognitive and WM dysfunction that may be highly relevant to understanding how dysfunction in short-term synaptic plasticity could fundamentally underlie schizophrenia pathophysiology.

Taken together, the results from studies in calcineurin-deficient mice indicate prominent deficits in WM and synaptic plasticity while basic synaptic properties appear largely intact. While this series of studies suggests calcineurin may play different roles in regulating synaptic plasticity in different brain regions (postsynaptic responses in the hippocampus vs. presynaptic neurotransmitter release in the PFC), their findings consistently point toward network-dysfunction in the context of alterations in synaptic plasticity. Although none of these studies provides an unequivocal causal link between synaptic plasticity deficits and the observed network dysfunction, these results nonetheless compellingly suggest that calcineurin deficiency or hypofunction in schizophrenia may lead to similar synaptic plasticity alterations that would lead to significant changes in the information processing performed by neural circuits.

### NRG1

Dysfunction in NRG1 signaling pathways has been implicated in the pathophysiology of a variety of neuropsychiatric diseases including schizophrenia (Stefansson et al., 2002; Bertram et al., 2007; Thomson et al., 2007; O'Donovan et al., 2008; Goes et al., 2009; Walker et al., 2010). The manifold NRG1 gene products are EGF-like ligands that signal through ERBB receptor tyrosine kinases—most relevantly through ERBB4—to regulate a large number of cellular processes including neuronal migration, synapse formation, myelination, synaptic transmission, and synaptic plasticity and both their normal signaling and altered signaling in neuropsychiatric diseases have been comprehensively reviewed recently (Mei and Nave, 2014). Although NRG1 has been implicated in an equivocal manner as a schizophrenia risk gene—primarily by human candidate gene studies—only a very small percentage of identified risk variants affect protein coding regions raising significant questions as to whether most identified non-coding variants lead to enhanced or decreased NRG1 signaling (Chen et al., 2006; Walss-Bass et al., 2006; Mei and Nave, 2014). As there is evidence supporting either enhanced or reduced NRG1 signaling in schizophrenia, no clear picture has emerged as to whether human NRG1 disease involves hypofunction or hyperfunction and this has led to the creation of distinct mouse models with opposing types of alterations in NRG1-ERBB signaling (Hashimoto et al., 2004; Petryshen et al., 2005; Hahn et al., 2006; Bertram et al., 2007; Parlapani et al., 2010; Shibuya et al., 2010). Despite the lack of a resolution of this very important issue that critically underlies the generation of valid genetic mouse models of disease, studies in both models of altered NRG1-ERBB signaling nonetheless reveal potential roles and sites of action for these molecules to alter synaptic plasticity and network function in schizophrenia.

Mouse models of both enhanced and reduced NRG1-ERBB signally display a variety of schizophrenia related behaviors. Mice with functional knockdown of NRG1 signaling show locomotor hyperactivity, impaired PPI of startle, impaired working memory, and abnormal social behaviors (Boucher et al., 2007; Chen et al., 2008; Ehrlichman et al., 2009; Shamir et al., 2012). Intriguingly, mice overexpressing components of NRG1-ERBB signaling display a similar pattern of behavioral alterations (Deakin et al., 2009, 2012; Yin et al., 2013; Luo et al., 2014). Importantly, at least some of these behaviors appear to result from semi-acute actions of NRG1-ERBB signaling—rather than complex developmental effects that could significantly impact neural circuit architecture—as perturbing or restoring expression acutely in adulthood can cause or partially reverse behavioral alterations, respectively (Yin et al., 2013; Agarwal et al., 2014; Luo et al., 2014). That many behavioral alterations appear due to acute actions of NRG1 suggests that underlying neural circuit dysfunction may be due to alterations in synaptic transmission and plasticity.

Until recently, the majority of studies exploring the impact of NRG1 signaling have focused upon the function of glutamatergic synapses where ERBB receptors co-localize with PSD95 suggesting a likely role in the regulation AMPA and NMDA receptors (Garcia et al., 2000; Huang et al., 2000; Hahn et al., 2006). Despite this fact, both pharmacological and genetic manipulation of NRG1 signaling does not appear to significantly affect basal glutamatergic synaptic transmission (Huang et al., 2000; Kwon et al., 2005; Bjarnadottir et al., 2007; Iyengar and Mott, 2008; Pitcher et al., 2008). Similarly—and in notable contrast to the findings from most other mouse models based upon schizophrenia risk genes—the preponderance of manipulations of NRG1 signaling have not revealed alterations in short-term glutamatergic synaptic plasticity suggesting NRG1 signaling may preferentially act postsynaptically at these synapses (Huang et al., 2000; Kwon et al., 2005; Iyengar and Mott, 2008; Pitcher et al., 2008; Chen et al., 2010; but see Bjarnadottir et al., 2007; Woo et al., 2007; Deakin et al., 2012). Conversely, NRG1 signaling has a significant effect upon both induction and expression of LTP with manipulations enhancing signaling suppressing LTP while reduced signaling facilitates LTP by removing an apparent tonic suppression (Huang et al., 2000; Kwon et al., 2005; Bjarnadottir et al., 2007; Pitcher et al., 2008; Chen et al., 2010; Agarwal et al., 2014, but see Deakin et al., 2012; Jiang et al., 2013). Although alterations in LTP at these synapses are usually interpreted as due to perturbations of glutamatergic synaptic physiology, complicating this interpretation of NRG1-ERBB signaling is accumulating evidence that NRG1 regulation of hippocampal LTP may be due to more powerful NRG1 facilitating actions at hippocampal GABA-ergic synapse (Chen et al., 2010; Shamir et al., 2012).

NRG1 signaling may particularly impact GABA-ergic transmission as its principal receptor, ErbB4, is selectively expressed in interneurons, particularly within the parvalbumin positive (PV) interneurons known to be fundamental to regulating synchronized network activity (Huang et al., 2000; Bartos et al., 2007; Fisahn et al., 2009; Vullhorst et al., 2009; Fazzari et al., 2010). Enhanced NRG1 signaling increases the amplitude of GABA-ergic synaptic events, most likely through a presynaptic mechanism that increases initial Pr to facilitate GABA release that further leads to altered short-term synaptic plasticity as manifested by enhanced short-term depression (Woo et al., 2007; Chen et al., 2010; Li et al., 2011). A potentially critical importance of the regulation of synaptic function and excitability specifically in PV interneurons by NRG1 is suggested by the alterations in network activity, behavior, and glutamatergic LTP that have been observed both in response to broad alterations in NRG1 signaling as well as when these manipulations are selectively confined to PV interneurons (Woo et al., 2007; Fisahn et al., 2009; Chen et al., 2010; Li et al., 2011; Andersson et al., 2012; Deakin et al., 2012; Shamir et al., 2012; Del Pino et al., 2013).

Overall, the findings from mouse models with a variety of manipulations of NRG1 signaling show prominent deficits in WM, synchronized neural circuit dysfunction likely due to PV interneuron dysfunction, and alterations in both long-term and short-term synaptic plasticity in the context of largely normal basal synaptic function. Furthermore, while on face the prevalence of LT vs. ST synaptic plasticity alterations observed seems at odds with results from mouse models based upon other schizophrenia risk genes, recent findings suggest that these LT plasticity alterations may in fact arise indirectly from altered presynaptic Pr in GABA-ergic neurons that display altered ST plasticity. Yet interpreting the causal impact of NRG1 signaling upon neural circuit function will require much further clarification because, in addition to its more acute effects upon synaptic transmission and synaptic plasticity, its developmental roles in neuronal migration, synapse formation, and myelination further suggests that altered NRG1 signaling in schizophrenia and other disease states may also lead to significant changes in neural circuit architecture.

### RGS4

Reduced expression of the “regulator of g-protein signaling” protein RGS4 has been implicated in schizophrenia. Normally, RGS4 acts a negative regulator of GPCR signaling due to its GAP-activity (GTPase-activating protein) that serves to terminate cycles a G-protein signaling by converting active Gα subunits to their inactive state (De Vries et al., 2000). A number of studies have indicated that RGS4 deficiency leads to enhanced GPCR signaling and that this may ultimately lead to a decrease in the probability of NT release from presynaptic terminals (decreased presynaptic Pr). Such an alteration in presynaptic function would be expected to lead changes in short-term synaptic plasticity that would affect the computation of neural circuits suggesting that such functional changes could contribute to the dysfunction in schizophrenia.

The paper that best describes the possible synaptic mechanism and impact of RGS4 deficiency upon presynaptic function, however, is a report that examines the effects of RGS2 deficiency—rather than RGS4 deficiency—upon synaptic function in cultured hippocampal neurons (Han et al., 2006). This study shows that presynaptic RGS2 deficiency permits enhanced presynaptic mGluR signaling that leads to increased inhibition of presynaptic voltage-gated Ca^2+^ channels (most likely through direct Gβγ inhibition of N-type or P/Q-type channels). The reduced activity of these presynaptic Ca^2+^ channels in RGS2-deficient synapses results in reduced neurotransmitter release in response to APs. When RGS2 is present in presynaptic terminals these hippocampal synapses are either depressing or non-facilitating by paired-pulse paradigms, indicative of a high presynaptic Pr. RGS2-deficient synapses, however, show paired-pulse facilitation suggesting that in the absence of RGS2 the presynaptic Pr is decreased. In additional support of this action at voltage-gated Ca^2+^ channels, RGS2-deficient synapses required more extracellular Ca^2+^ for the same degree of NT release as WT synapses yet both synapses showed indistinguishable NT release in response to hypertonic sucrose challenges. The results of this study indicate that RGS2 deficiency leads to changes in presynaptic function that result in changes in short-term synaptic plasticity and further suggests that a similar scenario may likely result in cases of RGS4 deficiency.

Although whether a similar mechanism of alteration in synaptic function occurs in cases of RGS4 deficiency has not been reported, two studies support this possibility by showing that RGS4 has a functional relationship with voltage-gated Ca^2+^ channel activity analogous to that of RGS2. Rather than exploring effects upon synaptic transmission, however, these two studies that look at RGS4 regulation of voltage-gated Ca^2+^ channels examine the resulting impact upon action potentials (Saugstad et al., 1998; Ding et al., 2006). One study found that conditions that enhanced expression of RGS4 in medium spiny neurons (MSNs) of the striatum led to the removal of muscarinic GPCR inhibition of voltage-gated Ca^2+^ channel activity. The enhanced voltage-gated Ca^2+^ channel activity that resulted from increases in RGS4 expression permitted enhanced Ca^2+^ currents during APs that recruited activation of Ca^2+^-activated K^+^-channels that served to regularize AP rhythms in these striatal neurons. The results of this paper indicate that RGS4 normally serves to reduce voltage-gated Ca^2+^ channel inhibition and suggest that RGS4 deficiency would lead to reduced voltage-gated Ca^2+^ activity.

A second study found that the normal suppression of Ca^2+^-activated K^+^ currents by mGluR activation in CA1 hippocampal pyramidal neurons could be nearly abolished by inclusion of RGS4 in the recording pipette (Saugstad et al., 1998). Although this study did not directly show that the RGS4 enhancement of Ca^2+^-activated K^+^ currents was due to removal of mGluR mediated inhibition of voltage-gated Ca^2+^ channels, this would seem to be the likely mechanism. Taken together, the results of these two studies supporting RGS4-mediated enhancement of voltage-gated Ca^2+^ channel activity, although they did not examine synaptic physiology, support the possibility that RGS4 deficiency could lead to a reduced voltage-gated Ca^2+^ channel activity in presynaptic terminals that would result in reduced NT release and altered short-term synaptic plasticity.

A final study directly explored the impact of RGS4 deficiency upon synaptic plasticity at MSNs in the striatum by employing RGS4-deficient mice (Lerner and Kreitzer, 2012). This study examined an endocannabinoid (eCB) mediated LTD (eCB-LTD) at MSN synapses that is dependent upon mGluR-Gq mediated generation of eCB in the post-synaptic neuron that travels to the presynaptic neuron to inhibit presynaptic NT release by lowering Pr. Normally this LTD pathway can be modulated by the activation of additional postsynaptic GPCRs, but in RGS4-deficient mice this modulation is absent. Through a series of elegant experiments this study showed that this GPCR modulation of LTD occurs through alterations in cAMP/PKA modulation of RGS4 activity that alters the ability of RGS4 to suppress mGluR-dependent production of the eCB retrograde messenger responsible for the LTD. Thus, although by a much more complicated mechanism than described for RGS2, RGS4 deficiency similarly results in alterations in presynaptic NT release probability (Pr). Further, although this study did not directly address changes in ST synaptic plasticity, the inability to modulate presynaptic Pr due to RGS4 deficiency would be predicted to lead to a decreased ability to modulate ST plasticity at this synapse as well.

Taken together, the results of these four studies suggest that the action of RGS4 should normally serve to increase presynaptic NT release probability and that RGS4 deficiency would lead to reductions in Pr causing reduced NT release. While only LT plasticity consequences have been directly explored in RGS4-deficient mice, the results of these studies supporting that presynaptic Pr is a likely target for RGS4 modulation strongly suggest that ST synaptic plasticity—and consequently neural circuit computation—would be strongly impacted by RGS4 deficiency in schizophrenia.

### Dysbindin

Reductions in dysbindin expression and function have been implicated in schizophrenia and results from a number of studies point toward a role for dysbindin in regulating presynaptic short-term plasticity by modulation of the probability of neurotransmitter release from synaptic terminals. Although a detailed understanding of dysbindin function remains to be determined, its expression in central synaptic terminals in the mammalian CNS and its association with synaptic vesicles suggests a possible role in the regulation of NT release (Benson et al., 2001; Talbot et al., 2006).

Perhaps the most informative study of dysbindin function was performed at the glutamatergic neuromuscular junction (NMJ) in *Drosophila* (Dickman and Davis, 2009), a system whose amenability to genetic manipulation permits rapid clarification of the detailed molecular mechanisms of gene action. This study demonstrated that presynaptic dysbindin deficiency led to reduced Ca^2+^-sensitivity of NT release, enhanced paired-pulse facilitation of post-synaptic responses, enhanced facilitation of post-synaptic responses during trains of stimuli, and a failure of homeostatic upregulation of the number of NT vesicles released per stimulus (quantal content) upon postsynaptic receptor blockade. Each of these results is consistent with a role of dysbindin in enhancing the Pr of presynaptic NT release (and dysbindin deficiency leading to reduced NT release), most likely through an action of dysbindin that enhances the Ca^2+^ sensitivity of the NT release machinery. This study convincingly illustrated that dysbindin deficiency altered ST plasticity by transforming a modestly facilitating synapse into a strongly facilitating synapse. This finding supports the possibility that dysbindin-deficient synapses in the mammalian CNS would have similar alterations in ST plasticity that would be expected to significantly alter synaptic and neural circuit computation.

A recent study of synaptic transmission onto layer V pyramidal neurons in the mPFC in dysbindin-null mice reported a number of findings consistent with the altered presynaptic function described at the Drosophila NMJ (Saggu et al., 2013). Reduced amplitudes of evoked EPSCs, reduced frequency of mEPSCs, a reduced RRP of synaptic vesicles, reduced SV cycling upon depolarization (as determined by FM1-43 studies), and decreased Ca^2+^ flux into synaptosomes upon depolarization all consistently point toward a decrease in the presynaptic Pr of NT release in dysbindin-deficient synapses mice. Furthermore, although not reaching statistical significance, this study performed assays to determine Pr more directly and showed a ~35% reduction in presynaptic Pr in dysbindin-deficient mice. Thus, although this study did not directly examine either short-term or long-term plasticity by traditional electrophysiological assays, taken together these findings strongly support a reduced presynaptic Pr due to dysbindin deficiency that would be expected to lead to alterations in short-term plasticity in neural circuits in the mammalian mPFC.

Despite the strong support for dysbindin deficiency leading to decreased presynaptic function through reduced Pr (Dickman and Davis, 2009; Saggu et al., 2013), a recent study in the hippocampus of dysbindin-deficient mice did not see alterations in ST plasticity (Tang et al., 2009). This study of Schaffer collateral input to CA1 in the hippocampus reported a nearly doubled magnitude of LTP at these synapses in dysbindin-deficient mice but both normal paired-pulse facilitation and normal LTD. Yet, taken together, these studies of synaptic function in dysbindin-deficient animals suggest that the most likely role of dysbindin is in regulating presynaptic NT release and that dysbindin deficiency leads to decreases in NT release by reducing presynaptic Pr. Furthermore, as presynaptic Pr is the prime determinant controlling short-term plasticity at synapses, dysbindin deficiency in schizophrenia would be predicted to most significantly alter short-term plasticity and lead to alterations in neural circuit computation that could underlie disease symptomology.

### AKT1

Reductions in AKT1 expression and function have been implicated in schizophrenia in a number of recent studies (Emamian et al., 2004; Zhao et al., 2006; Balu et al., 2012). AKT is a protein kinase that has many cytosolic protein targets in a variety signaling pathways both in the CNS as well as in non-neuronal tissues (Yang et al., 2004). AKT1-deficient mice show alterations in the dendritic architecture of mPFC pyramidal neurons as well as working memory deficits that are believed to be representative of analogous deficits found in schizophrenia (Lai et al., 2006). Although the role AKT1 in neuronal and synaptic function remains under-explored, two studies in animal models with reduced AKT1 expression have revealed alterations in synaptic plasticity (Guo and Zhong, 2006; Balu et al., 2012).

While neither of these two studies provides a clear mechanistic explanation of the plasticity alterations observed in AKT1-deficient animals, the study of the glutamatergic synapse at the NMJ of *Drosophila* provides a more comprehensive analysis (Guo and Zhong, 2006). In this study the authors describe a novel long-term depression (LTD) evoked by high frequency stimulation that quantal analysis revealed to be presynaptic in origin as the quantal size remained unchanged but the quantal content (reflecting the number of released neurotransmitter vesicles per stimulated event) was reduced. Although basic NMJ responses were normal in AKT mutant flies, this novel LTD was significantly reduced in mutant flies with low expression of AKT while normal LTD could be rescued by acute over-expression of WT AKT. Despite the evidence of a presynaptic locus for this LTD, in AKT mutant flies with deficient LTD, measures of baseline presynaptic function such as short-term depression, short-term facilitation, and PTP were found to be normal. Given that these results support that the observed LTD in WT flies was due to reduced presynaptic Pr and that all forms of baseline presynaptic function in AKT-deficient flies were normal—including inferred presynaptic Pr—suggests a role of AKT in the induction of changes in presynaptic Pr during LTD protocols that is reduced or absent in AKT-deficient flies. Interestingly, such a mechanistic deficiency is somewhat similar to the deficient control of presynaptic Pr upon eCB-LTD induction in the striatum of RGS4-deficient mice where RGS deficiency impacts the ability to modulate presynaptic Pr (Lerner and Kreitzer, 2012). Regardless of the details of the mechanism underlying deficient LTD at the NMJ in AKT-deficient flies, an inability to appropriately reduce presynaptic Pr by long-term plasticity mechanisms would consequently lead to an inability to alter presynaptic short-term plasticity normally at AKT-deficient synapses.

In a separate study in AKT1-deficient mice deficits in long-term plasticity were observed at multiple synapses in the hippocampus (Balu et al., 2012). AKT1-deficient mice showed deficits in LTP in CA1 upon Schaffer collateral stimulation (SC-CA1) and deficits in LTP in the dentate gyrus upon medial perforant path stimulation (EC-GC). At both of these sites short-term plasticity of synaptic responses during the tetanus were normal in AKT1-deficient mice suggesting no major alteration in baseline presynaptic short-term plasticity mechanisms. The post-tetanic short-term potentiation, however, although normal at SC-CA1 synapses, was completely absent at EC-GC synapses suggesting a more severe plasticity deficit at the synapses onto granule cells in the dentate gyrus. Despite the profound deficits in synaptic plasticity described in AKT1-deficient mice in this report, given that the mechanisms underlying these deficits were not further experimentally pursued it is difficult to speculate upon the precise role of AKT1 function at these synapses.

Taken together the results of these two studies in AKT-deficient animals indicate that AKT1 may play an significant role in regulating long-term synaptic plasticity and that AKT1 deficiency in schizophrenia may result in similar long-term plasticity alterations that could contribute to disease symptomology.

## Autism

With the exception of schizophrenia, perhaps the most relevant studies evaluating synaptic plasticity that employ genetic mouse-models based upon strong candidate risk genes in human neuropsychiatric disease have been carried out in models of autism. Autism spectrum disorder (ASD) is a heterogeneous neurodevelopmental with an approximate 1% prevalence in the population that has a heritability supporting a strong genetic component (Abrahams and Geschwind, 2008). ASD is symptomology is characterized by abnormal reciprocal social interactions, impaired communication (particularly involving language but also non-verbal), and repetitive behaviors (American Psychiatric Association, 2013). Among the diverse array of autism risk genes that have been identified both from syndromes with autistic features as well as though genome analyses of large patient populations are a significant number of genes known to affect synaptic function and plasticity (Zoghbi, 2003; Abrahams and Geschwind, 2008; Südhof, 2008; Silverman et al., 2010; Gauthier and Rouleau, 2012; Iossifov et al., 2012; Neale et al., 2012; O'Roak et al., 2012; Sanders et al., 2012). And, in contrast genetic mouse models of schizophrenia which predominantly display selective alterations in short-term synaptic plasticity, mouse models based on ASD risk genes have shown a more balanced combination of both short- and long-term synaptic plasticity alterations (Arguello and Gogos, 2012).

Functional imaging studies in autism overall support that long-range connections between brain regions (such as from higher-order association areas to the frontal lobe) show reduced functional connectivity while local microcircuitry and short-range connections show evidence of enhanced connectivity (Mundy, 2003; Belmonte et al., 2004; Geschwind and Levitt, 2007; Markram and Markram, 2010; Ecker and Murphy, 2014). Assessment of network activity in autism with EEG and MEG has shown reduction of local microcircuit activity evidenced by reduced gamma oscillations at rest and during sensory processing and facial presentation tasks (Wilson et al., 2007; Sun et al., 2012; but see Orekhova et al., 2007). Furthermore, long-range connectivity between brain regions appears impaired as, despite increased strength of theta rhythms locally, coherence of these rhythms between brain regions was reduced (Murias et al., 2007). That some of these network alterations have also been seen in unaffected relatives of autistics suggests that both local and long-range network dysfunction may represent significant intermediate endophenotypes (Rojas et al., 2011). Finally, *in vivo* studies of long-term plasticity have indicated that at least a subset of autistics show enhanced LTP- and LTD-like phenomena that are consistent with theories of hyper-plasticity in autism (Markram and Markram, 2010; Oberman et al., 2010).

Although a number of mouse models of autism based on human genetic findings have been made, neuronal and synaptic function have been most thoroughly explored in mice modeled upon Rett syndrome (MECP2 gene deletion) and fragile X syndrome (FMR1 gene deletion). While both of these human disorders are syndromic in nature and manifest a variety of clinical features in addition to ASD symptomology, important insights about synaptic plasticity alterations underlying autism symptomology can still be gleaned from these mouse models despite these potentially complicating factors. Both Fmr1 and Mecp2 null mice demonstrate deficits in long-term memory and learning that are consistent with the possibility of an underlying deficit in long-term synaptic plasticity (Moretti et al., 2006; MacLeod et al., 2010). While there are no reports on working memory (MW)—a short-term memory process thought to rely largely upon short-term synaptic plasticity—in Mecp2 null mice, MW memory may be deficient in Fmr1 null mice although reports are conflicting (Van Dam et al., 2000; Yan et al., 2004; Mongillo et al., 2008; Udagawa et al., 2013).

Both Mecp2 and Fmr1 null mice show alterations in presynaptic short-term plasticity. Decreased paired-pulse facilitation observed in Mecp2 null mice suggests increased initial Pr for neurotransmitter release that shifts synaptic function in the direction of short-term depression (Moretti et al., 2006; Weng et al., 2011). These mice have also shown reduced PTP, a more sustained form of short-term plasticity induced by high-frequency activity (Weng et al., 2011). In Fmr1 null mice, although paired-pulse facilitation is normal, these mice showed strongly increased augmentation—a longer-lasting component of short-term plasticity—and reduced short-term depression that is entirely due to presynaptic mechanisms and were at least partially attributable to enhanced presynaptic Ca^2+^ influx due to AP broadening and increases in the readily releasable pool of NT vesicles during high frequency activity (Deng et al., 2011, 2013; Wang et al., 2014). These changes also resulted in changes in synaptic information processing as there was a significant enhancement of responses during natural stimulus trains that were most notable during high-frequency epochs of activity (Deng et al., 2011).

Both Mecp2 and Fmr1 null mice show alterations in long-term plasticity. MeCP2 null mice have been shown to have impaired hippocampal LTP and LTD in the hippocampus and impaired LTP in motor and sensory cortex (Asaka et al., 2006; Moretti et al., 2006; Weng et al., 2011). Although Fmr1 null mice display normal hippocampal LTP they have impaired neocortical LTP and show enhanced LTD in both the hippocampus and cerebellum (Godfraind et al., 1996; Paradee et al., 1999; Huber et al., 2002; Li et al., 2002; Koekkoek et al., 2005; Larson et al., 2005; Hayashi et al., 2007).

The limited studies of network activation in mouse models of autism suggest enhanced activity in local microcircuitry. Drug-induced gamma oscillations are enhanced in Mecp2 null mice and, although gamma oscillations have not been assessed in Fmr1 null mice, these mice also display local network dysfunction in the form of enhanced up-states during slow wave oscillations (Hays et al., 2011; McLeod et al., 2013). These enhancements in local network activity in mouse models of autism are consistent with some reports of enhanced high-frequency oscillations observed in humans studies (Orekhova et al., 2007). Coupled with the findings in human studies, these complementary findings in mouse models of autism suggest that significant alterations in both long-term and short-term synaptic plasticity may likely be responsible for the information processing and behavioral dysfunction present in the human disease.

## A strategy to dissecting neural circuit dysfunction in genetic mouse models of neuropsychiatric disease

While a small number of etiologically valid genetic mouse models of schizophrenia and autism have begun to enable exploration of the underlying dysfunction in neural circuits and synaptic plasticity in these diseases, similar progress for most other neuropsychiatric diseases has been limited to date as equally robust predisposing genetic variants necessary to recapitulate disease in animal models have yet to be identified for these diseases. Yet it is both the complex nature of the genetic risk of neuropsychiatric disease as well as our current symptoms-based vantage point that continue to present a number of challenging issues that more broadly complicate and confound the strategic approach to unraveling root causes underlying neural circuit dysfunction in genetic mouse models of neuropsychiatric diseases.

### Issue—genetic architecture

Genomic studies of neuropsychiatric disease have identified a surprisingly large and diverse array of risk genes. Despite this genetic heterogeneity, the fact that identified risk genes are preferentially involved in synaptic function, growth, and maintenance as well as axonal guidance suggests that genetic risk factors for neuropsychiatric disease may converge upon the common substrate of neural circuit function (Walsh et al., 2008; Glessner et al., 2009; Guilmatre et al., 2009; Akil et al., 2010; Insel et al., 2010; Gilman et al., 2011; O'Dushlaine et al., 2011; Sanders et al., 2011). In addition, despite heterogeneity, a small number of rare but highly penetrant genetic risk variants have been identified that reliably lead to disease that is clinically indistinguishable from the majority of disease cases (Farooqi and O'Rahilly, 2005; Peltonen et al., 2006). Although these highly penetrant genetic variants are usually only responsible for a very small percentage of cases of the broader disease, their high reliability to cause disease renders them invaluable, essential tools in creating mouse models that faithfully recapitulate disease. While a small number of such high-risk genetic variants have been identified in cases of schizophrenia, autism, and AD that have enabled the creation of reliable disease models in mice, for many common neuropsychiatric diseases—e.g., major depression, bipolar disorder, anxiety disorders, attention-deficit hyperactivity disorder—similarly high-risk genetic variants that reliably lead to human disease have yet to be identified thus leaving valid genetic mouse models of these diseases currently out of reach (Pulver et al., 1994; Hardy, 1997; Murphy et al., 1999; Millar et al., 2000; Blackwood et al., 2001; Hatton et al., 2006; Clifford et al., 2007; Ramocki et al., 2009; Vassos et al., 2010; Neul, 2012). While the genetic selective pressures—such as the strong selection pressures against retaining risk genes for socially debilitating and early-onset diseases like autism and schizophrenia that largely preclude successful reproductive transmission—that shape the genetic architecture of disease may be substantially different for diseases such as major depression, it nonetheless seems reasonable to posit (and hope) that high-risk genetic variants for these diseases in fact exist yet have eluded detection and remain to be found. Based upon these considerations, creating reliable genetic mouse models of disease must begin with the identification of highly penetrant genetic variants and in the case of neuropsychiatric diseases in which such genetic variants remain to be identified our understanding of the details of neural circuit dysfunction in these diseases likely must patiently await this first crucial step.

### Issue—overlapping disease phenomenology

Further complicating the understanding of the causality between genes and disease, genomic studies have revealed a surprising overlapping genetic risk among multiple neuropsychiatric diseases that raises significant questions as to the validity of the boundaries between these diseases (Craddock and Owen, 2005; Craddock et al., 2006; Friedman et al., 2008; Guilmatre et al., 2009; McCarthy et al., 2009; O'Donovan et al., 2009; Williams et al., 2010; Smoller et al., 2013). That is, in many cases the same genes implicated in increased risk for one neuropsychiatric disease have been shown to predispose to increased risk for multiple other distinct neuropsychiatric diseases. Furthermore, although some genetic variants appear to lead to narrower, better-defined clinical phenotypes, genomic studies have also revealed that in some cases the same genetic variant can be found in patients with different clinical diagnoses (St Clair et al., 1990; Hatton et al., 2006; Bijlsma et al., 2009; McCarthy et al., 2009; Ramocki et al., 2009; Shi et al., 2009; Wang et al., 2009; Karayiorgou et al., 2010; Shinawi et al., 2010). The potential significance of these results is further supported by heritability studies that have shown that within family pedigrees the presence of one neuropsychiatric illness is frequently associated with cross-vulnerability to other neuropsychiatric illnesses (Biederman et al., 1991; Maier et al., 1993; Lichtenstein et al., 2009b, 2010; Rommelse et al., 2010; Sullivan et al., 2012). Furthermore, beyond considering these concerns of genetic promiscuity among distinct neuropsychiatric diseases further suggests that beyond choosing highly penetrant genetic variants, the clearest path to understanding distinct diseases through genetic mouse models may further require carefully choosing among genetic variants for those associated with narrow, well-defined clinical presentations.

Beyond these concerns, however, the additional overlapping symptomology and overlapping responsiveness to medication found between some diseases raises the legitimate possibility that the boundaries between “distinct” diseases may be inherently blurred due to a true shared causation (Milberger et al., 1995; Citrome et al., 2005; Lake and Hurwitz, 2007; O'Donovan et al., 2009; Sinzig et al., 2009; Ivleva et al., 2010; Gros et al., 2012; Zbozinek et al., 2012). For example, while NMDA receptor hypofunction has been implicate in the causation of schizophrenia, recent studies in rodent models support NMDA receptor hypofuction in autism suggesting the possibility of a continuum of related dysfunction between and among distinct diseases (Mohn et al., 1999; Gandal et al., 2012; Saunders et al., 2012; Schmeisser et al., 2012; Won et al., 2012). Taken together, these multiple issues of overlapping disease phenomenology suggest the possibility that the traditional diagnostic criteria used to create distinct boundaries between diseases based upon behavioral manifestations—while clinically useful in disease management—may not be ideally suited as a scientific framework from which to determine underlying disease mechanisms which may instead require the development of more refined and quantifiable measures targeted at directly assessing endpoints related to brain function including the function of neural circuits (Geyer, 2006; Gould and Gottesman, 2006; Benes, 2007; Psychiatric GWAS Consortium Coordinating Committee, 2009; Luck et al., 2011; Smoller et al., 2013).

### Issue—alternative measures of function

Unlike traditional physiological diseases such as cardiovascular disease, diabetes, or obesity, the understanding of neuropsychiatric disease processes is currently hampered by the underdevelopment and underutilization of analytic measures that provide simple yet precise quantitative readouts of the function and status of the underlying physiological systems that directly contribute to disease pathogenesis (Gould and Gottesman, 2006). For example, psychiatric diagnosis and evaluation as put forth by the DSM-V is based upon a checklist of behavioral signs and symptoms that are considered either present or absent in a binary manner and are far removed from any direct quantitative measure of underlying brain or neural circuit function (American Psychiatric Association, 2013). Furthermore, most current evaluative measures in neuropsychiatric disease are inherently complex—e.g., evaluation of complex phenomena such as social function, judgment, and language—and do not obviously point to precisely circumscribed loci of neural circuit dysfunction. Due to this complexity, these measures—while both interesting and clinically relevant in terms of monitoring the progression and the functional impact of disease—are not terribly useful as scientific bases for a reductionist approach aimed at revealing fundamental disease mechanisms (Insel et al., 2010). Moreover, many of these complex manifestations seem to be uniquely human in nature do not appear to present obvious translatable equivalents that could readily permit correlative evaluation in mouse models. These considerations highlight the intense need in neuropsychiatric diseases for the development of more simplified and precise parameter assays that directly and quantitatively report upon underlying pathophysiological processes such as neural circuit dysfunction (Gould and Gottesman, 2006; Akil et al., 2010; Insel et al., 2010).

Analysis of disease related endophenotypes may represent the most effective strategy to provide the simplified and precisely quantitative functional measures required to efficiently dissect fundamental disease mechanisms. Endophenotypes are relatively simple, quantifiable, and heritable characteristics present in both individuals diagnosed with disease and in unaffected members within their familial pedigrees and are therefore believed to represent disease vulnerability traits (Gould and Gottesman, 2006). Compared to “top level” symptoms in neuropsychiatric disease that often appear to be uniquely human in nature without clear parallels in mouse models, the more rudimentary nature of endophenotypes present the further advantage that their assays are frequently readily translatable into mouse models (Geyer, 2008; Ecker and Murphy, 2014). In consensus recognition of the need for more quantifiable measures in neuropsychiatric disease, an initial, though partial, effort to address these issues strategically has recently begun to be laid out by the NIMH in the form of their proposed Research Domain Criteria (RDoC) project initiative.

While assessment of endophenotypes frequently provides little direct clinical utility, their quantification is scientifically valuable as endophenotypes are believed to represent the most fundamental consequences of the basic pathological processes underlying the more complex manifestations neuropsychiatric diseases. From the standpoint of assessing suspected neural circuit dysfunction in neuropsychiatric disease, particularly appealing among endophenotypes are the neurophysiological measures which quantify rather stereotyped responses to very simplified stimuli (Turetsky et al., 2007; Luck et al., 2011). These assays, through assessing the dysfunction of simplified neural circuits involved with very rudimentary information processing, present an effective and strategic platform from which to reveal the cellular and molecular details of the neuronal and synaptic alterations underlying neural circuit dysfunction in neuropsychiatric disease. To better appreciate the refined analytical power and broad utility such assays present for guiding a detailed mechanistic understanding of neural circuit dysfunction in neuropsychiatric disease, it is useful to specifically examine the strategic approach to understanding neural circuit dysfunction in mouse models of schizophrenia.

### Optimizing the approach to neural circuit analysis: schizophrenia as example

Currently the most prevalent approach to understanding neural circuit dysfunction in genetic mouse models of schizophrenia has involved assessing working memory deficits in these animals coupled with electrophysiological recordings from the underlying brain regions believed to contribute most to working memory performance. While not as striking as the positive symptomology of schizophrenia, the pervasive cognitive deficits in schizophrenia have presented a tractable experimental endophenotype for examining disease-relevant alterations in behavior and neural circuit function in genetic mouse models (Glahn et al., 2003; Heinrichs, 2005; Saperstein et al., 2006; Forbes et al., 2009; Arguello and Gogos, 2010, 2012). As such, assessment of working memory in genetic mouse models of schizophrenia has been commonly employed and any working memory deficits detected have been interpreted as reflecting dysfunction of neural circuits in either the hippocampus or the medial prefrontal cortex (Arguello and Gogos, 2010, 2012). Motivated by such findings these brain regions have then been subsequently probed more directly with electrophysiological assays in attempts to detect and localize neuronal dysfunction within specific neurons or synapses and to determine the precise mechanisms underlying this dysfunction and this approach has successfully revealed—consistent with WM deficits—prominent alterations in short-term synaptic plasticity (Zeng et al., 2001; Kvajo et al., 2008, 2011; Mongillo et al., 2008; Jentsch et al., 2009; Sigurdsson et al., 2010; Cottrell et al., 2013; Fénelon et al., 2013).

Despite the strong validity, disease-relevance, and translational potential of working memory tasks as a framework from which to assess and reverse neuronal and synaptic dysfunction in mouse models, the inherent complexity of such tasks limits their potential to identify and precisely localize underlying neuronal dysfunction (Arguello and Gogos, 2010, 2012). Specifically, during a spatial working memory task information processing in disparate brain regions involving sensory, motor, motivational, volitional, appetitive, and fear and anxiety related neural circuits is likely influencing behavioral performance in addition to the neural circuit activation within the mPFC and hippocampus (Gisquet-Verrier and Delatour, 2006; McNab and Klingberg, 2008; Edin et al., 2009; Baddeley, 2012).

As the ultimate goal of investigations in genetic mouse models of neuropsychiatric disease is to reveal the precise mechanisms of neuronal and synaptic dysfunction underlying the neural circuit dysfunction in these diseases, the optimal assays of *in vivo* neural circuit function should ideally be as simple and uncomplicated as possible such that any dysfunction observed can be confidently and unambiguously attributed to a distinct neural circuit or brain region that can then be directly targeted with invasive, high-resolution electrophysiological assays. Although finding a functional measure that can be purely attributed to a single, well-defined neural circuitry is a non-trivial hurdle, a number of schizophrenia-related neurophysiological endophenotypes involving sensory evoked responses may come the closest to fitting this requirement (Turetsky et al., 2007; Thaker, 2008). While the study of the neural circuits underlying these endophenotypes has yet to be aggressively pursued, due to their exquisite simplicity these endophenotypes may represent the most tractable and efficient analytical path toward understanding the discrete neuronal and synaptic mechanisms underlying neural circuit dysfunction in well-defined and valid genetic animal models of schizophrenia (Goff et al., 1980; Javitt et al., 1994; Freedman et al., 1996; Swerdlow et al., 2001; Grunwald et al., 2003; Kumari et al., 2003; Schall et al., 2003; Luck et al., 2011).

Specifically, among the many neurophysiological assays showing characteristic alterations in schizophrenics, the sensory evoked response endophenotypes involving PPI of acoustic startle, P50 auditory evoked potential suppression (P50), and acoustic mismatch negativity (MMN) appear to reflect information processing abnormalities within relatively simple and isolated neural circuits (Clements et al., 1998; Bramon et al., 2004; Light and Braff, 2005; Swerdlow et al., 2006; Brockhaus-Dumke et al., 2008). From a reductionist point of view, the most compelling characteristic of these three endophenotype assays is that they involve neural circuit processing that is either entirely or largely preattentive—and thus nearly reflexive—in nature (Naatanen, 1992). These measures thus appear to represent read-outs of rudimentary neural circuit computation with minimal contaminating influences from other neural circuitry that might otherwise confound interpretation of results (Naatanen, 2003; Braff and Light, 2004). As such, these assays of neural circuit function that are largely devoid of the complications of attentional, motivational, or cognitive control may be optimal assays of neural circuit function in mouse models of schizophrenia—through use of analogous assays in rodents that have been developed for each of these measures—as they examine hard-wired, relatively inflexible computational elements that present a greatly simplified system in which to explore the discrete neuronal and synaptic mechanisms underlying neural circuit dysfunction (Swerdlow et al., 2001; Gould and Gottesman, 2006; Metzger et al., 2007; Geyer, 2008; Javitt et al., 2008; Ehrlichman et al., 2009; Luck et al., 2011). Furthermore, as these neurophysiological assays employ either pairs or trains of acoustic stimuli delivered at very brief intervals, these assays bear a striking resemblance to electrophysiological assays that employ similarly brief intervals of stimulation to determine short-term plasticity properties of synapses suggesting the possibility that abnormalities observed in neurophysiological assays may reflect underlying alterations in short-term synaptic plasticity (Freedman et al., 1996; Garrido et al., 2009; Blundell et al., 2010). In summary, while no neural circuit exists in isolation from the influence of other circuits within the brain, the neurophysiological assays of preattentive schizophrenia endophenotypes that activate rudimentary neural circuits come close to this ideal and may represent the optimal assays to guide the elucidation of the synaptic and neuronal mechanisms underlying neural circuit dysfunction in valid genetic mouse models of schizophrenia.

## Concluding remarks

The progress in unraveling the mechanisms underlying neural circuit dysfunction in human neuropsychiatric diseases has been enormously accelerated by the recent advances in human genomics that have enabled the identification of an abundance of candidate genetic risk variants that may be the responsible agents for causing disease symptomology. Specifically, the identification of highly penetrant risk genes for schizophrenia and autism has led the creation of etiologically valid mouse models for these diseases that have enabled detailed investigations directed at identifying synaptic, neuronal, and neural circuit dysfunction. Studies in these mouse models have revealed both neural circuit dysfunction as well as a variety of synaptic plasticity alterations that may be responsible for this dysfunction given the critical role of synaptic plasticity in sculpting information flow within neural circuits. Overall, while mouse models of schizophrenia have shown a preponderance of short-term plasticity alterations consistent with disease symptomology, mouse models of autism appear to present a more balanced combination of both short-term and long-term plasticity alterations that may be consistent with the more profound dysfunction present in the clinical picture of this spectrum of disorders.

These initial successes with schizophrenia and autism strongly suggests that, despite the failure to date to identify highly penetrant genetic risk variants for many of the more common neuropsychiatric diseases such as anxiety disorders and major depression, this strategic approach may represent the necessary pathway forward for these diseases as well. Yet, beyond negotiating the complexities of the genetic architecture of neuropsychiatric diseases, in order to effectively identify neural circuit dysfunction and resolve the underlying neuronal and synaptic mechanisms that lead to it, additional considerations need to be thoughtfully addressed. As a symptomology-based approach to neuropsychiatric disease has revealed significant overlapping disease phenomenology, dissecting the causes of unique forms of neural circuit dysfunction underlying disease will likely require further identification and utilization of more simplified, quantifiable endophenotypes that examine precisely defined parameters more directly and more closely related to alterations in specific brain regions and functions. In addition to the advantage that assays of such endophenotypes are readily translatable to assess neural circuit dysfunction in mouse models of disease, assessing the genetic risk variants contributing to individual endophenotypes—compared to the global risk for disease based upon symptomology—should facilitate dissecting disease mechanisms due to the likely identification of a much smaller and more manageable number of genes responsible for dysfunction. Finally, although our current understanding of even the most fundamental aspects of normal neural circuit function remain rudimentary, the continuing technical advances in *in vivo* imaging, electrophysiology, and optogenetics are beginning to help reveal new levels of details of normal neural circuit that will enable more sophisticated assessment of neural circuit function in disease. Taking these considerations together, the strategic approach to understanding neural circuit dysfunction in neuropsychiatric disease is rapidly evolving on multiple technical fronts and now seems poised to shortly make major revelations regarding the mechanisms of neural circuit dysfunction in these diseases.

### Conflict of interest statement

The authors declare that the research was conducted in the absence of any commercial or financial relationships that could be construed as a potential conflict of interest.
